# The association between alcohol consumption, cardiac biomarkers, left atrial size and re-ablation in patients with atrial fibrillation referred for catheter ablation

**DOI:** 10.1371/journal.pone.0215121

**Published:** 2019-04-10

**Authors:** Neshro Barmano, Emmanouil Charitakis, Robert Kronstrand, Ulla Walfridsson, Jan-Erik Karlsson, Håkan Walfridsson, Fredrik H. Nystrom

**Affiliations:** 1 Department of Medical and Health Sciences, Linköping University, Linköping, Sweden; 2 Department of Internal Medicine, County Hospital Ryhov, Jönköping, Sweden; 3 Department of Cardiology, Linköping University Hospital, Linköping, Sweden; 4 National Board of Forensic Medicine, Linköping, Sweden; 5 Primary Health Care Centre Centrum, Norrköping, Sweden; Scuola Superiore Sant'Anna, ITALY

## Abstract

**Background:**

Information on alcohol consumption in patients undergoing radiofrequency ablation (RFA) of atrial fibrillation (AF) is often limited by the reliance on self-reports. The aim of this study was to describe the long-term alcohol consumption, measured as ethyl glucuronide in hair (hEtG), in patients undergoing RFA due to AF, and to examine potential associations with cardiac biomarkers, left atrial size and re-ablation within one year after the initial RFA.

**Methods:**

The amount of hEtG was measured in patients referred for RFA, and a cut-off of 7 pg/mg was used. N-terminal pro B-type natriuretic peptide (NT-proBNP) and the mid-regional fragment of pro atrial natriuretic peptide (MR-proANP) were examined and maximum left atrium volume index (LAVI) was measured. The number of re-ablations was examined up to one year after the initial RFA. Analyses were stratified by gender, and adjusted for age, systolic blood pressure, body mass index, presence of heart failure and heart rhythm for analyses regarding NT-proBNP, MR-proANP and LAVI and heart rhythm being replaced by type of AF for analyses regarding re-ablation.

**Results:**

In total, 192 patients were included in the study. Median (25th– 75^th^ percentile) NT-proBNP in men with hEtG ≥ 7 vs. < 7 pg/mg was 250 (96–695) vs. 130 (49–346) pg/ml (p = 0.010), and in women it was 230 (125–480) vs. 230 (125–910) pg/ml (p = 0.810). Median MR-proANP in men with hEtG ≥ 7 vs. < 7 pg/mg was 142 (100–224) vs. 117 (83–179) pmol/l (p = 0.120) and in women it was 139 (112–206) vs. 153 (93–249) pmol/l (p = 0.965). The median of maximum LAVI was 30.1 (26.7–33.9) vs. 25.8 (21.4–32.0) ml/m^2^ (p = 0.017) in men, and 25.0 (18.9–29.6) vs. 25.7 (21.7–34.6) ml/m^2^ (p = 0.438) in women, with hEtG ≥ 7 vs. < 7 pg/ml, respectively. Adjusted analyses showed similar results, except for MR-proANP turning out significant in men with hEtG ≥ 7 vs. < 7 pg/mg (p = 0.047). The odds ratio of having a re-ablation was 3.5 (95% CI 1.3–9.6, p = 0.017) in men with hEtG ≥ 7 vs. < 7 pg/mg, while there was no significant difference in women.

**Conclusions:**

In male patients with AF and hEtG ≥ 7 pg/mg, NT-proBNP and MR-proANP were higher, LA volumes larger, and there was a higher rate of re-ablations, as compared to men with hEtG < 7 pg/mg. This implies that men with an alcohol consumption corresponding to an hEtG-value ≥ 7, have a higher risk for LA remodelling that could potentially lead to a deterioration of the AF situation.

## Introduction

Atrial fibrillation (AF) is the most common cardiac arrhythmia, with a prevalence of approximately 3% in the Swedish population [1]. The prevalence of AF increases with age [1], and due to an ageing population, the prevalence of patients with AF is expected to rise. AF is associated with heart failure, disabling symptoms, decreased health-related quality of life (HRQoL), increased mortality, and risk of ischaemic stroke [1, 2].

Restoring and maintaining sinus rhythm (SR) is an integral part of AF treatment [2]. In the acute setting, electrical cardioversion or anti-arrhythmic drugs (AAD) can convert AF into SR. In the long term, maintaining SR can be achieved through continuous medication with AAD, or through catheter ablation with isolation of the pulmonary veins, achieved with radiofrequency energy or cryoballoon ablation [2]. Radiofrequency ablation (RFA) of AF is more effective than AAD in maintaining SR, and can nowadays be considered as first-line therapy in selected patients [2].

Although there has been a substantial increase in AF research, previous studies have mainly focused on treatment [3]. Disease prevention is now increasingly attracting more attention and risk factor modification is recognised as the fourth pillar, besides stroke prevention, rate and rhythm control, in the management of AF [4–6]. Established risk factors for the development of AF, of which some are modifiable and some are not, include age, gender, heart failure, valvular heart disease, myocardial infarction, hypertension, diabetes mellitus, obesity, and smoking [4]. Inflammatory and neurohumoral biomarkers, obstructive sleep apnoea, metabolic syndrome and prolonged endurance training are also associated with increased risk of AF [4, 7].

High alcohol consumption is yet another modifiable risk factor for AF [8], and recently it has been found that even light to moderate intake of alcohol is associated with increased risk of developing AF [9]. However, the mechanisms by which alcohol consumption is linked to an increased risk of AF remain unclear. Several possible mechanisms have been suggested, such as direct cardiotoxicity, induction of hyper adrenergic activity, alterations in oxidative stress, conduction abnormalities and atrial remodelling [10, 11]. Furthermore, reports from studies on alcohol consumption in patients undergoing RFA of AF are scant [11, 12], and so far have been limited by the reliance on self-reports, in which patients are prone to underreport the actual intake [13]. To the best of our knowledge, no previous study has assessed long-term alcohol consumption with any laboratory measures in patients undergoing RFA of AF.

The aim of this study was to describe long-term alcohol consumption, evaluated with an objective marker, in a population with AF referred for RFA, and to examine the associations of both measured and self-reported alcohol consumption with cardiac biomarkers, ejection fraction, left atrial (LA) size and re-ablation.

## Methods

### Study design and population

This was an observational, single-centre study based on data from the SMURF (Symptom burden, Metabolic profile, Ultrasound findings, Rhythm, neurohormonal activation, haemodynamics and health-related quality of life in patients with atrial Fibrillation) study [14]. The SMURF study was conducted between January 2012 and April 2014. Patients referred for RFA due to AF to the University Hospital in Linköping, Sweden, were considered for participation. The inclusion criteria were: 1) Age ≥ 18 years with paroxysmal or persistent AF, 2) Patients referred for first time RFA, and 3) Patients with sufficient knowledge of the Swedish language to fill out the study questionnaires.

Exclusion criteria were: 1) Patients who had previously undergone catheter or surgical AF ablation, 2) Patients with previous or planned heart surgery, 3) Patients with left ventricular ejection fraction (EF) <35%, and 4) Patients with acute coronary syndrome during the past three months.

### Informed consent and ethical considerations

The Regional Ethical Review Board in Linköping, Sweden, approved the study (Dnr 2011/40-31, 2012/226-32). All patients gave their written consent and the study complied with the Declaration of Helsinki[15].

### Study flow chart

The protocol of the SMURF study has been published previously [16]. In brief, enrolled patients were subjected to a full baseline evaluation including medical history, self-reported alcohol consumption, physical examination and a 12-lead electrocardiogram (ECG). Questionnaires were filled out and a transthoracic echocardiography (TTE) was performed the day before RFA. The sagittal abdominal diameter was measured and a tuft of hair was cut, close to the scalp, for the analysis of ethyl glucuronide (hEtG). All patients were catheterised according to clinical routine at the ablation date. Before the ablation, blood samples for the analysis of biomarkers, described below, were drawn from the femoral vein. The ablation procedure is described below. The number of re-ablations was examined up to 12 months after the initial RFA.

### Ablation procedure

Procedures were performed under conscious sedation, using propofol and remifentanil. Two transseptal vascular sheaths were inserted through the right femoral vein into the left atrium and perfused using heparinized saline with irrigation rate 2ml/h. Heparin was administered to maintain an activated clotting time of >350 seconds throughout the procedure. Mapping and ablation was performed using an open-irrigated catheter (ThermoCool^TM^, Biosense Webster, Diamond Bar, CA, USA) and the computer-based mapping system CARTO^TM^ (Biosense Webster, Diamond Bar, CA, USA). A 7-F, 20-pole circumferential diagnostic catheter was used for assessment of pulmonary vein activation and isolation. Radiofrequency energy was delivered in a power-controlled mode with maximum energy setting 35 W at an irrigation rate of 17–30 mL/min. Endpoint of all ablation procedures was electrical disconnection of all pulmonary veins by antral ablation, verified by entry- and exit block in all pulmonary veins. In patients with persistent AF, additional ablation in order to create LA lines was at the discretion of the operator and verified by pacing maneuvers.

### Assessment of alcohol consumption

#### Ethyl glucuronide in hair

In man, most of the ingested ethanol is oxidised to acetaldehyde through alcohol dehydrogenase. A very small portion,<0.1%, is conjugated with activated glucuronic acid by UDP-glucuronosyl transferases, forming ethyl glucuronide, detectable in hair [17]. The total coefficient of variation (CV) for hEtG was 16% at 7 pg/mg, 8.3% at 33 pg/mg, and 6.2% at 267 pg/mg hair. In this study, hair samples were collected as close as possible to the scalp and the proximal 3 cm were used for estimation of hEtG as described earlier [17], corresponding to the alcohol consumption during the last three months. According to the Society of Hair Testing, an hEtG concentration ≥ 7 pg/mg is indicative of repeated alcohol consumption and provides evidence to refute a claim of abstinence while concentrations ≥30 pg EtG/mg strongly suggest excessive/chronic alcohol consumption [18]. In this study, we analysed possible differences between those above and those below the cut-off level of 7 pg/mg.

#### Self-reported alcohol consumption

Patients were interviewed about their weekly alcohol consumption, and the reported amount of alcohol was translated into units. In this study, one unit corresponded to eight grams of alcohol, ingested in the form of beer, wine or spirits. High consumption was defined as more than 14 or 9 units/week in men and women [19], respectively, and low consumption as consumption below this level, including teetotallers.

### Cardiac biomarker measurements and other laboratory tests

The concentrations of the N-terminal pro B-type natriuretic peptide (NT-proBNP), the mid-regional fragment of pro atrial natriuretic peptide (MR-proANP), cholesterol, high density lipoprotein (HDL) and triglycerides (TG) were measured as previously described [20]. Alanine aminotransferase (ALT), aspartate aminotransferase (AST) and glutamyl transferase (GT) were measured according to clinical routine. Low density lipoprotein (LDL) cholesterol was calculated using Friedewald’s formula: *LDL-cholesterol* = *total-cholesterol – HDL-cholesterol* – (0.45*xTG*). The total CV for NT-proBNP was 4.6% at 426.5 pg/mL and 3.2% at 2308 pg/mL [20]. The CV for MR-proANP according to the manufacturer was ≤5% for concentrations between 10 and 20 pmol/L, <3.5% for concentrations between 20 and 1000 pmol/L, and <3.5% for concentrations over 1000 pmol/L [20].

### Echocardiography

All patients underwent TTE prior to RFA. GE Vivid 7 or GE Vivid E9 system (GE Healthcare, Horten, Norway) were utilised. Left ventricular EF was calculated by the biplane Simpson’s method. LA volume was measured using the biplane area-length method and was corrected for body surface area to obtain the LA volume index (LAVI).

### Statistics

Normally distributed continuous variables were expressed as means ± standard deviation (SD) and tested with the independent t-test between two groups. Non-normally distributed variables were expressed as medians with 25th to 75th percentiles within brackets and tested with the Mann-Whitney U-test between two groups. Categorical data were presented as counts with percentages within brackets and tested with Chi-square test between groups. Spearman´s correlation coefficient was used to assess the correlation between self-reported alcohol consumption and hEtG. Multiple linear regression analysis was performed in order to correct for gender when evaluating the relationship between analysable hEtG and HDL, and CHA_2_DS_2_VASc score, respectively. The dependent variable was HDL and CHA_2_DS_2_VASc score, respectively, with analysable hEtG (yes/no) as an independent variable and gender (male/female) as a covariate. Multiple linear regression analysis was also performed in order to evaluate the predictive role of gender and sagittal abdominal diameter on HDL in this population. The dependent variable was HDL and the independent variables were sagittal abdominal diameter, gender and the use of statins. Analyses of differences in cardiac biomarkers and echocardiographic measurements between two groups, based on the hEtG cut-off level of 7 pg/mg, and on self-report, i.e. high vs. low consumption, were stratified according to gender, due to the fact that cosmetic hair treatment can affect the hEtG-analysis. They were also adjusted for age, systolic blood pressure, body mass index (BMI), heart failure (yes/no) and actual heart rhythm (SR/AF), through multiple linear regression analysis. In this analysis, logarithmic transformation was used for NT-proBNP and MR-proANP in order to achieve normal distribution. Binary logistic regression analysis was performed, with adjustment for the above mentioned co-variates, except for heart rhythm being replaced by AF type (paroxysmal/persistent), in order to analyse the association between alcohol consumption and re-ablation. All reported p-values were two-sided and a p-value <0.05 was considered statistically significant. The analyses were performed using the SPSS 24.0 (SPSS, Chicago, IL, USA).

## Results

### Baseline characteristics

**Fig 1:**
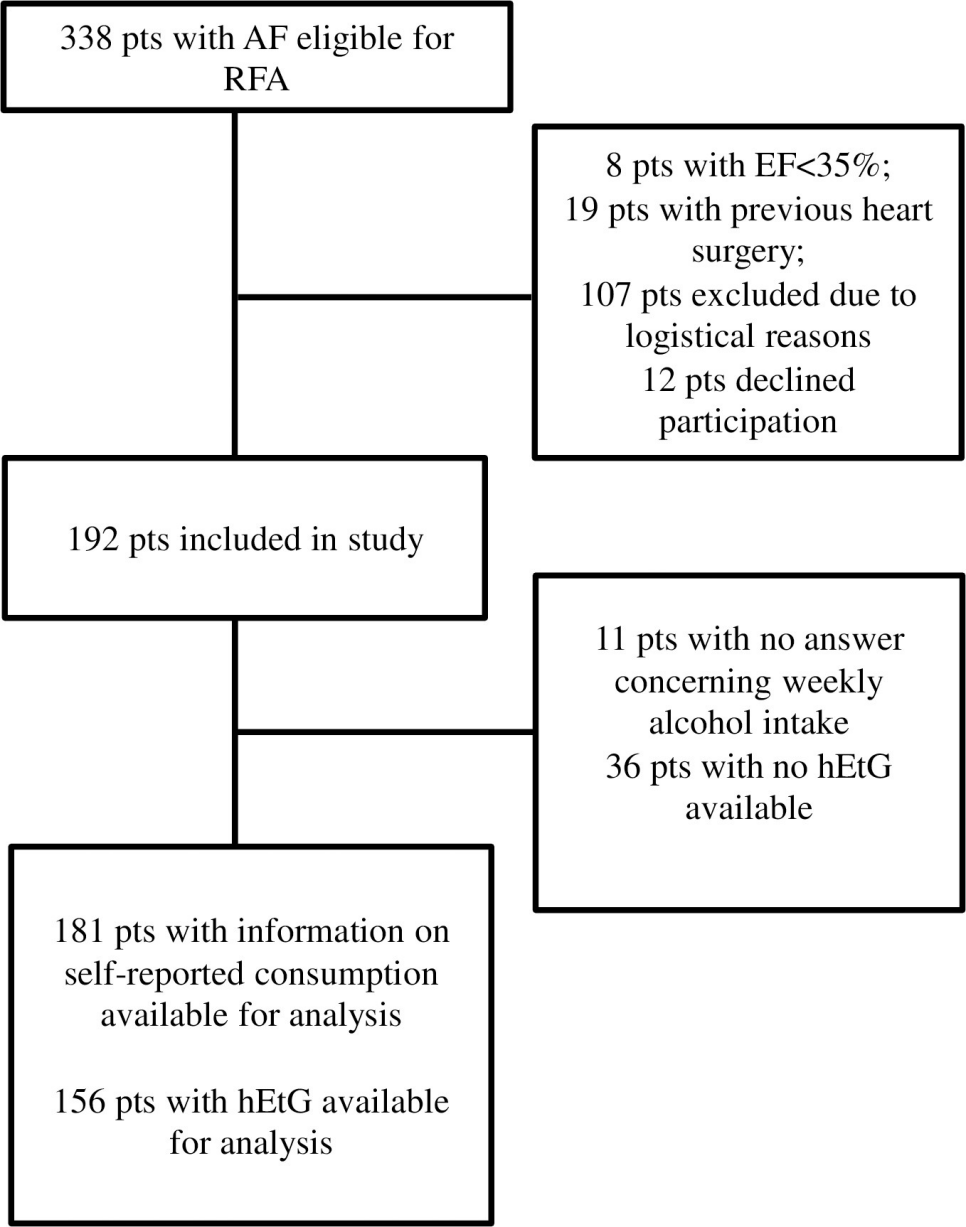
Study inclusion flowchart. AF: atrial fibrillation; EF: ejection fraction; hEtG: hair ethyl glucuronide; pts: patients; RFA: radiofrequency ablation.

In total, 192 patients with AF referred to the Department of Cardiology, University Hospital in Linköping, Sweden, were included. Information about self-reported alcohol consumption was available for 181 (94%) and hEtG was analysable for 156 (81%) patients (Fig 1). There was a statistically significant correlation between self-reported alcohol consumption and hEtG (r = 0.63, p<0.001). The baseline characteristics are presented in Table 1. Of those with non-analysable hEtG, one was a woman whose sample analysis failed for technical reasons, while the remaining 35 patients were men without, or with too short, hair. The group with non-analysable hEtG had lower HDL and CHA_2_DS_2_VASc scores (Table 1). However, this was probably due to differences in gender rather than to whether hEtG was analysable or not, since when correcting for gender, the analysis showed that analysable hEtG was not correlated to HDL (p = 0.438, model: R^2^ = 0.208, F = 24.759, p <0.001), nor to CHA_2_DS_2_VASc score (p = 0.749, model: R^2^ = 0.179, F = 20.658, p <0.001), but rather gender was (p<0.001 for both HDL and CHA_2_DS_2_VASc scores). As seen in Table 1, men were younger, had a larger sagittal abdominal diameter, higher AST, ALT, GT, and TG as well as lower HDL and CHA_2_DS_2_VASc scores than women, and were more likely to have non-analysable hEtG. Analyses regarding the predictive role of gender and sagittal abdominal diameter on HDL showed that sagittal abdominal diameter (inversely related, p < 0.001) and gender (p <0.001), but not the use of statins (p = 0.349), were related to HDL (model R^2^ = 0.266, F = 22.243, p <0.001). Baseline characteristics according to hEtG levels and according to self-reported alcohol consumption are also presented in Table 1.

**Table 1:** Baseline characteristics.

**Variables **	**Total study population (n = 192)**	**hEtG analysable (n = 156)**	**hEtG not analysable (n = 36)**	**p-value**	**Men (n = 136)**	**Women (n = 56)**	**p-value**	**hEtG <7 pg/mg (n = 113)**	**hEtG ≥7 pg/mg (n = 43)**	**p-value**	**Low consumption (n = 159)**	**High consumption (n = 22)**	**p-value**
***Characteristics and concomitant diseases***													
Age	60.5±10.2	61.1±10.1	57.7±10.2	0.075	58.7±10.4	64.8±8.5	<0.001	61±11	60±9	0.615	60±11	61±8	0.651
Female gender	56 (29%)	55 (35%)	1 (3%)	<0.001	-	-	-	45 (40%)	10 (23%)	0.053	48 (30%)	7 (32%)	0.876
Sagittal abdominal diameter (cm)	24.6±4.4	24.4±4.2	25.5±5.0	0.158	25.1±4.4	23.4±4.2	0.014	23.8±4.3	25.8±3.8	0.008	24.5±4.4	24.9±3.9	0.725
BMI (kg/m^2^)	28.0±4.2	27.9±4.2	28.3±4.4	0.573	28.2±4.3	27.3±4.1	0.171	27.7±3.4	28.9±3.7	0.064	27.8±4.3	28.6±4.0	0.414
Current smokers	5 (3%)	5 (3%)	0	§	3 (2%)	2 (4%)	§	4 (4%)	1 (2%)	§	4 (3%)	1 (5%)	§
Previous smokers	93 (48%)	79 (51%)	14 (39%)	0.203	64 (47%)	29 (52%)	0.551	49 (43%)	30 (70%)	0.003	72 (45%)	16 (73%)	0.016
Hypertension	82 (43%)	69 (44%)	13 (36%)	0.375	57 (42%)	25 (45%)	0.728	50 (44%)	19 (44%)	0.994	67 (42%)	11 (50%)	0.485
Diabetes mellitus	16 (8%)	12 (8%)	4 (11%)	§	10 (7%)	6 (11%)	0.444	9 (8%)	3 (7%)	0.836	16 (10%)	0 (0%)	§
Vascular disease	13 (7%)	10 (6%)	3 (8%)	§	9 (7%)	4 (7%)	§	6 (5%)	4 (9%)	0.363	8 (5%)	2 (9%)	§
Heart failure	18 (9%)	16 (10%)	2 (6%)	§	15 (11%)	3 (5%)	0.220	12 (11%)	4 (9%)	0.809	15 (9%)	2 (9%)	§
CKD (GFR<60mL/min/1.73 m^2^)	40 (21%)	31 (20%)	9 (25%)	0.495	29 (21%)	11 (20%)	0.794	20 (18%)	11 (26%)	0.270	32 (20%)	3 (14%)	§
Stroke/TIA	19 (10%)	14 (9%)	5 (14%)	0.373	12 (9%)	7 (13%)	0.438	10 (9%)	4 (9%)	0.930	13 (8%)	4 (18%)	0.132
CHA_2_DS_2_VASc	2 (0–3)	2 (1–3)	1 (0–2)	0.048	1 (0–2)	3 (2–3)	<0.001	2 (1–3)	1 (1–3)	0.542	2 (0–3)	2 (1–3)	0.297
Systolic blood pressure (mmHg)	146±20	146±20	147±21	0.662	146±20	145±22	0.662	146±20	146±20	0.988	126±18	121±20	0.168
Diastolic blood pressure (mmHg)	90±11	90±11	90±11	0.864	91±11	89±11	0.414	90±12	91±9	0.457	74±12	75±11	0.741
AST (μkat/L)	0.46 (0.39–0.52)	0.45 (0.38–0.52)	0.47 (0.40–0.57)	0.378	0.47 (0.40–0.54)	0.43 (0.34–0.49)	0.012	0.44 (0.37–0.52)	0.46 (0.41–0.51)	0.382	0.46 (0.35–0.59)	0.44 (0.39–0.55)	0.681
ALT (μkat/L)	0.46 (0.36–0.59)	0.46 (0.35–0.60)	0.43 (0.0.38–0.59)	0.985	0.47 (0.38–0.61)	0.37 (0.30–0.51)	<0.001	0.46 (0.34–0.60)	0.47 (0.37–0.62)	0.276	0.46 (0.35–0.59)	0.41 (0.34–0.62)	0.670
GT (μkat/L)	0.46 (0.34–0.74)	0.45 (0.32–0.75	0.47 (0.38–0.74)	0.432	0.50 (0.38–0.78)	0.37 (0.29–0.57)	0.002	0.42 (0.30–0.62)	0.60 (0.42–1.40)	<0.001	0.45 (0.34–0.74)	0.52 (0.34–1.15)	0.261
Total cholesterol (mmol/L)	5.0±1.2	5.0±1.2	4.8±1.2	0.397	4.9±1.1	5.1±1.3	0.288	5.0±1.2	4.9±1.1	0.735	4.9±1.2	5.2±1.0	0.313
LDL (mmol/L)	3.2±1.0	3.2±1.0	3.1±1.1	0.649	3.2±1.0	3.2±1.1	0.938	3.2±1.1	3.1±1.0	0.443	3.2±1.1	3.3±0.9	0.458
HDL (mmol/L)	1.2±0.34	1.3±0.4	1.1±0.3	0.016	1.1±0.27	1.5±0.4	<0.001	1.3±0.3	1.2±0.4	0.550	1.2±0.4	1.3±0.3	0.486
TG (mmol/L)	1.2±0.60	1.2±0.6	1.3±0.6	0.401	1.3±0.6	1.0±0.5	0.006	1.1±0.5	1.3±0.8	0.111	1.2±0.6	1.3±0.6	0.594
***Alcohol consumption***													
Self-reported consumption (units/week)	4 (1–9)	4 (1–9)	3 (0–8)	0.488	5 (1–9)	3 (0–6)	0.041	2.5 (0–6)	9 (6–15)	<0.001	3 (0–6)	18 (13–24)	<0.001
Ethyl glucuronide concentration (pg/mg)	0 (0–8)	-	-	-	0 (0–8.2)	0 (0–5.6)	0.213	0 (0–0)	15 (10–26)	<0.001	0 (0–7.7)	3.8 (0–8.3)	0.421

Continuous data are presented as means with standard deviation for normally distributed variables or as median values with 25^th^ to 75^th^ percentile within brackets for non-normally distributed variables. Categorical data are presented as counts with percent values within brackets. Baseline data are presented for all available patients, and for groups according to whether hEtG was analysable or not, gender, hEtG <7 vs. ≥7 and low vs. high self-reported alcohol consumption. p-values for the differences between groups are calculated with the student’s t-test for normally distributed continuous variables, the Mann-Whitney U-test for non-normally distributed continuous variables and with the Chi-square test for categorical variables. AF: atrial fibrillation; ALT: alanine aminotransferase; AST: aspartate aminotransferase; BMI: body mass index; CHA_2_DS_2_ VASc: congestive heart failure, hypertension, age ≥ 75, diabetes, stroke/TIA, vascular disease, age 65–74, sex category; CKD: chronic kidney disease; GT: glutamyl transferase; GFR: glomerular filtration rate; hEtG: hair ethyl glucuronide; HDL: high density lipoprotein; LD: lactate dehydrogenase; LDL; low density lipoprotein; TG: triglycerides; TIA: transient ischaemic attack.

§p-values not reported due to no fulfilment of assumptions for Chi-square test

### Alcohol consumption

#### Ethyl glucuronide in hair

Nine (6%) patients had hEtG-values ≥ 30 pg/mg, while 88 patients (56%) had an hEtG-value of 0 pg/mg. The group with hEtG ≥ 7 pg/mg were more likely to be previous smokers, had larger sagittal abdominal diameter, higher GT levels and a higher self-reported alcohol consumption compared to the group with hEtG < 7 pg/mg (Table 1).

#### Self-reported alcohol consumption

The median self-reported alcohol consumption was 4 (1–9) units/week and men reported higher intake than women (Table 1). The group with self-reported high alcohol consumption were more likely to be previous smokers (Table 1).

### Associations between alcohol consumption and cardiac biomarkers

#### Ethyl glucuronide in hair

**Fig 2:**
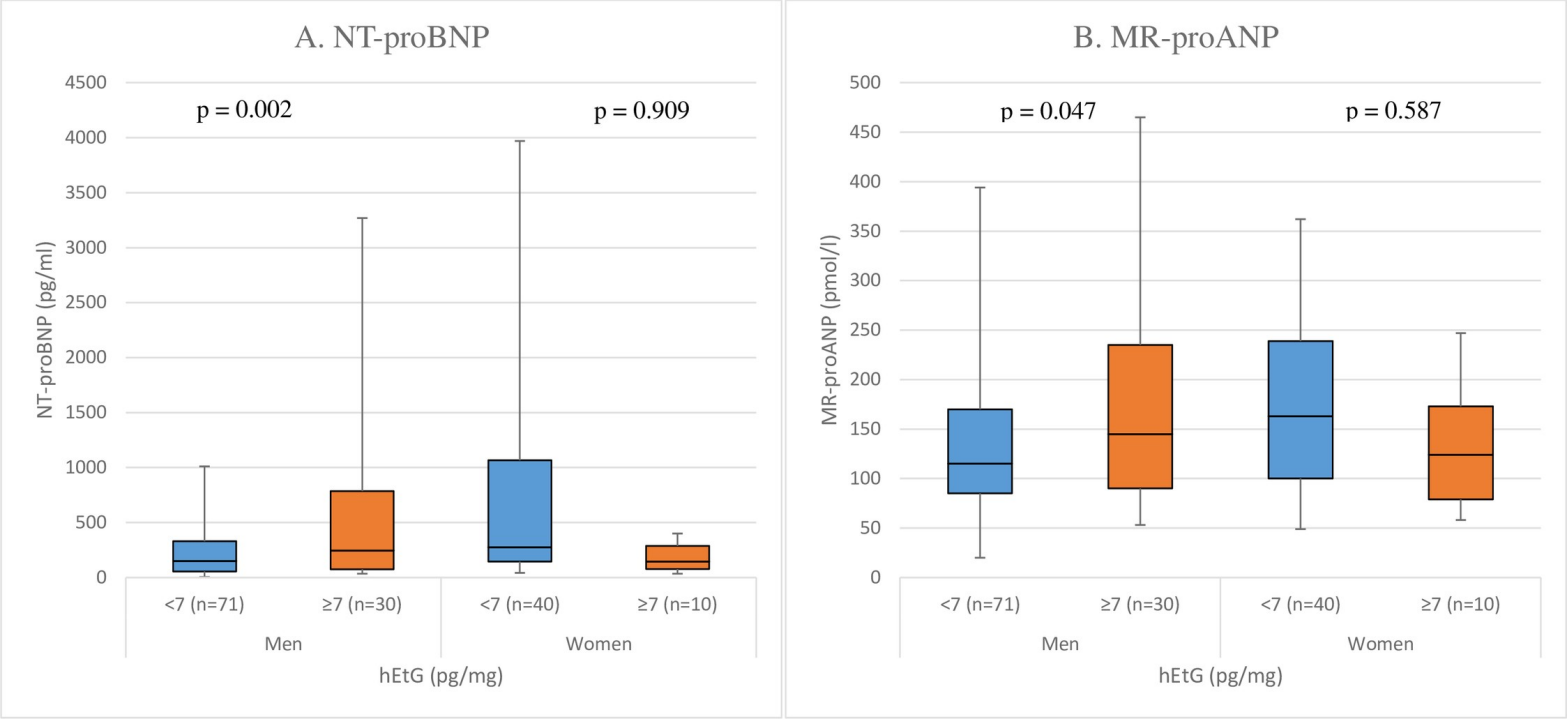
Cardiac biomarkers in different hair ethyl glucuronide groups in men and women. The p-values were obtained through multiple linear regression analysis, using age, systolic blood pressure, body mass index (BMI), heart failure (yes/no) and actual heart rhythm (SR/AF) as co-variates. hEtG: Ethyl glucuronide in hair; MR-proANP: mid-regional fragment of the N-terminal precursor of atrial natriuretic peptide; NT-proBNP: N-terminal fragment of prodromal B-type natriuretic peptide.

NT-proBNP was significantly higher in men with hEtG ≥ 7 pg/mg as compared to men with hEtG < 7 pg/mg (Table 2). Adjusted analysis showed significant difference in MR-proANP in men with hEtG ≥ 7 pg/mg vs. < 7pg/mg, while no significant differences in either of the cardiac biomarkers were found in women (Table 2 and Fig 2).

**Table 2:** Cardiac biomarkers and echocardiographic measurements according to different levels of ethyl glucuronide in hair.

	**Total study population**				**Men**				**Women**			
	hEtG <7 pg/mg (n = 113)	hEtG ≥ 7 pg/mg (n = 43)	Crude p-value	Adjusted p-value	hEtG <7 pg/mg (n = 68)	hEtG ≥ 7 pg/mg (n = 33)	Crude p-value	Adjusted p-value	hEtG <7 pg/mg (n = 45)	hEtG ≥ 7 pg/mg (n = 10)	Crude p-value	Adjusted p-value
***Cardiac biomarkers***												
NT-proBNP (pg/ml)	170 (66–489)	250 (110–660)	0.109	0.031	130 (49–346)	250 (96–695)	0.010	0.002	230 (125–910)	230 (125–480)	0.810	0.909
MR-proANP (pmol/l)	133 (88–194)	140 (101–221)	0.229	0.055	117 (83–179)	142 (100–224)	0.120	0.047	153 (93–249)	139 (112–206)	0.965	0.587
***Echocardiographic measurements***												
EF (%)	57±9	55±8	0.238	0.454	56±10	53±8	0.113	0.409	58±9	62±4	0.086	0.743
Max LAVI (ml/m^2^)	25.8 (21.6–32.7)	29.1 (23.7–32.2)	0.103	0.317	25.8 (21.4–32.0)	30.1 (26.7–33.9)	0.017	0.024	25.7 (21.7–34.6)	25.0 (18.9–29.6)	0.438	0.079
Min LAVI (ml/m^2^)	14.9 (11.1–19.4)	16.2 (11.1–21.4)	0.190	0.368	14.6 (10.5–19.3)	17.5 (12.0–22.7)	0.029	0.038	15.0 (11.9–19.9)	12.1 (9.4–19.1)	0.228	0.097

Normally distributed continuous data are presented as means with standard deviation and non-normally distributed continuous data as median values with 25^th^ to 75^th^ percentiles within brackets. Crude p-values for differences between groups were calculated with the student’s t-test for normally distributed continuous variables and the Mann-Whitney U-test for non-normally distributed continuous variables. Adjustment for age, systolic BP, BMI, heart failure and actual heart rhythm, was made using multiple linear regression analysis, and using the logarithmic transformation for NT-proBNP and MR-proANP in order to achieve normal distribution. BMI: body mass index; BP: blood pressure; EF: ejection fraction; hEtG: hair ethyl glucuronide; LA: left atrium; LAVI: left atrium volume index; MR-proANP: mid-regional fragment of the N-terminal precursor of atrial natriuretic peptide; NT-proBNP: N-terminal fragment of prodromal B-type natriuretic peptide

#### Self-reported alcohol consumption

There were no significant differences in NT-proBNP or MR-proANP between self-reported high and low consumption, either in the analysis of the entire study population, or according to gender (Table 3).

**Table 3:** Cardiac biomarkers and echocardiographic measurements according to different levels of self-reported alcohol consumption.

	**Total study population**				**Men**				**Women**			
	Low consumption (n = 159)	High consumption (n = 22)	Crude p-value	Adjusted p-value	Low consumption (n = 111)	High consumption (n = 15)	Crude p-value	Adjusted p-value	Low consumption (n = 48)	High consumption (n = 7)	Crude p-value	Adjusted p-value
***Cardiac biomarkers***												
NT-proBNP (pg/ml)	170 (67–529)	223 (133–465)	0.344	0.477	150 (58–455)	220 (100–450)	0.390	0.257	240 (130–868)	320 (150–1628)	0.605	0.531
MR-proANP (pmol/l)	129 (89–188)	140 (101–217)	0.460	0.713	121 (83–179)	140 (107–200)	0.386	0.254	163 (98–203)	139 (78–309)	0.791	0.411
***Echocardiographic measurements***												
EF (%)	56.9±8.7	55.5±9.5	0.502	0.347	55.7±8,9	56.1±8.8	0.883	0.840	59.6±7.6	54.4±11.5	0.119	0.163
Max LAVI (ml/m^2^)	26.4 (22.4–32.7)	29.4 (23.4–33.0)	0.506	0.754	27.2 (22.8–32.3)	29.1 (23.3–33.4)	0.417	0.359	25.9 (21.7–33.8)	30.0 (22.1–34.2)	0.905	0.730
Min LAVI (ml/m^2^)	14.9 (11.0–19.7)	15.0 (10.7–20.3)	0.931	0.633	14.9 (10.9–19.3)	15.0 (10.5–20.7)	0.887	0.942	14.7 (11.3–20.2)	15.6 (11.8–23.5)	0.933	0.640

Normally distributed continuous data are presented as means with standard deviation and non-normally distributed continuous data as median values with 25^th^ to 75^th^ percentiles within brackets. Crude p-values for differences between groups were calculated with the student’s t-test for normally distributed continuous variables and the Mann-Whitney U-test for non-normally distributed continuous variables. Adjustment for age, systolic BP, BMI, heart failure and actual heart rhythm, was made using multiple linear regression analysis, and using the logarithmic transformation for NT-proBNP and MR-proANP in order to achieve normal distribution. High consumption was defined as more than 14 or 9 units/week (1 unit = 8 grams alcohol) in men and women, respectively, and low consumption as consumption below this level, including teetotallers. BMI: body mass index; BP: blood pressure; EF: ejection fraction; LAVI: left atrium volume index; MR-proANP: mid-regional fragment of the N-terminal precursor of atrial natriuretic peptide; NT-proBNP: N-terminal fragment of prodromal B-type natriuretic peptide

### Associations between alcohol consumption and echocardiographic measurements

#### Ethyl glucuronide in hair

No significant differences were found when analysing the entire study population (Table 2). When divided according to gender, maximum and minimum LAVI were higher in men with hEtG ≥ 7 pg/mg (Table 2). No such corresponding differences were found in women (Table 2).

#### Self-reported alcohol consumption

No significant differences in echocardiographic measurements were observed between the high and low self-reported alcohol consumption groups (Table 3).

### Association between alcohol consumption and re-ablation

#### Ethyl glucuronide in hair

Re-ablation was performed in 58 (30%) patients. Crude analysis showed that there was a trend for more frequent re-ablation in men with hEtG ≥ 7 pg/mg vs. < 7 pg /mg (14 (42%) vs. 16 (24%), p = 0.051) while no difference was found in women (3 (30%) vs. 13 (29%), p = 0.944). Adjusted analysis showed a significant correlation between hEtG and re-ablation in men (OR 3.5; 95% CI 1.3–9.6; p = 0.017) but not in women (OR 0.60; 95% CI 0.1–3.1; p = 0.541).

#### Self-reported alcohol consumption

There was no significant difference between the high and low self-reported alcohol consumption groups in the frequency of re-ablation, not in crude analysis (men: 7 (47%) vs. 32 (29%), p = 0.161; women: 2 (29%) vs. 14 (29%), p = 0.974), nor in adjusted analysis (men: OR 2.7; 95% CI 0.8–8.9, p = 0.09; women: OR 1.3; 95% CI 0.2–10.0, p = 0.77).

## Discussion

To the best of our knowledge, this is the first study investigating the association between an objective marker of alcohol consumption with cardiac biomarkers and echocardiographic measurements as well as re-ablation in patients undergoing RFA due to AF. We found that men with hEtG ≥ 7 pg/mg had higher NT-proBNP and MR-proANP, larger LA volumes and a higher rate of re-ablations than men with hEtG < 7 pg/mg, while no such findings were present in women.

### Alcohol consumption and baseline characteristics

hEtG ≥ 7 pg/mg was associated with higher GT values and previous smoking, as expected. Although a significantly higher HDL also would have been expected in the group with hEtG ≥ 7 pg/mg, this was not the case in this study. Regarding alcohol and heart disease, much attention has been paid to the possible positive effects of light to moderate alcohol consumption on cardiovascular disease, especially coronary artery disease [21], partly explained by the association between alcohol consumption and elevated HDL levels [22]. However, in a study by Hung et al., alcohol consumption was associated with low HDL levels, which was thought to be explained by the higher body mass index in subjects with light to moderate alcohol consumption [23]. In this study, alcohol consumption corresponding to an hEtG level ≥ 7 pg/mg was also associated with a larger sagittal abdominal diameter, which could have outweighed the possible differences in HDL between the two hEtG-groups.

Although there was a significant correlation between self-reported alcohol consumption and hEtG, there were several variables that differed significantly between the two hEtG-groups, while previous smoking was the only variable that differed significantly between the two self-reported alcohol consumption groups. Possible reasons for this might be that hEtG better reflects long-term alcohol consumption than data acquired from interviews concerning alcohol intake, but could also be due to some subjects not being completely honest about their actual alcohol consumption.

### Associations between alcohol consumption, cardiac biomarkers, echocardiographic measurements and re-ablation

#### Cardiac biomarkers

hEtG ≥7 pg/mg was associated with higher NT-proBNP and MR-proANP concentrations in men, consistent with a previous large study by Djousse et al, in which there was a dose-response related positive correlation between reported alcohol consumption and ANP [24]. In comparison to previous studies which have shown that excessive alcohol consumption is related to elevated levels of B-type natriuretic peptide [25, 26], our study shows that even moderate consumption, as indicated by an hEtG-value ≥7 pg/mg, is associated with increased levels of NT-proBNP.

No significant differences in cardiac biomarkers were present in the female group. There are several possible explanations for the gender-related differences. One explanation could be that women more often use cosmetic hair treatments and thermal hair straightening tools, which can affect the hEtG results, and the test could therefore be more insensitive in women. Another possible explanation might be the small number of women, rendering the analyses in women underpowered.

#### Echocardiographic measurements

Men with hEtG ≥ 7 pg/mg had larger LA volumes than men with hEtG < 7 pg/mg. This is consistent with the study conducted by McManus et al., in which the relationship between alcohol consumption and left atrial size in 5220 offspring and original Framingham Heart Study participants was studied [27]. The study showed that every additional 10 g of alcohol per day was associated with a 0.16 mm larger left atrial dimension and subsequent incident AF. Similarly, Hung et al. have shown that light to moderate intake of alcohol in a dose-dependent manner is related to larger LA volumes as well as impaired LA strain [23].

### Re-ablation

Men with hEtG ≥ 7 pg/mg had a 3-fold increased odds of having a re-ablation, compared to men with hEtG < 7 pg/mg. This is consistent with the study by Takigawa et al, who showed a strong association between alcohol consumption and AF recurrence after the initial RFA[12]. Qiao et al. also showed an association between alcohol consumption and unfavourable ablation outcomes[11]. In contrast to those studies, this study also used an objective marker of alcohol consumption in addition to self-reported alcohol intake.

The pathophysiology behind the negative effects of alcohol on the heart is complex and several possible mechanisms for the increased risk of AF have been suggested [10, 11]. On the other hand, studies have also shown positive cardiac effects of moderate alcohol intake [28]. If, and at which point in the individual’s lifetime of alcohol intake, negative characteristics appear is highly individual [29]. Before a left ventricular systolic dysfunction is evident, subclinical alterations in myocardial contractility may occur [23]. On the atrial level this is supported by studies that show a possibly dose-response relationship between alcohol consumption and atrial remodelling [11]. Furthermore, studies have shown an inverse relationship between the degree of fibrosis as assessed by magnetic resonance imaging, and LA strain and strain rate [30]. Impaired systolic LA mechanics, as indicated by LA enlargement and impaired strain, might thus be a measure of the subclinical alterations associated with alcohol consumption. Although this study does not explain the exact link between alcohol consumption and AF, it strengthens the conclusion drawn by McManus et al., that “LA enlargement is a key intermediate phenotype along the causal pathway to AF”. These facts might explain previous findings that even moderate intake of alcohol is associated with the risk of AF [9], and that impaired systolic LA mechanics can predict success in maintaining SR after cardioversion and RFA [31, 32], and could thus potentially serve as an explanation for more frequent re-ablations in men with hEtG ≥ 7 pg/mg that we found.

Although AF prevention in the form of assessment of modifiable risk factors, such as alcohol consumption, is a cornerstone in the management of AF, research in this field has been scant. However, the field is an emerging research area, where recent studies have highlighted the importance of risk factor management in AF. Studies have demonstrated improvement in AF burden and outcomes post RFA via weight management and cardiorespiratory fitness, which might also improve HRQoL [3, 14]. In the ARREST-AF study, treatment of several risk factors including alcohol abstinence or reduction of alcohol intake to below 30 g per week, were included as parts of an aggressive cardiovascular risk factor management programme, leading to improved AF-related outcomes [5]. However, it is impossible to distinguish the effect of reduced alcohol consumption from the other measures taken in the study.

### Limitations

We acknowledge that our study was a single-centre observational cohort study of a moderate size. Furthermore, in line with previous studies, in which social drinkers often have unmeasurable hEtG results [33, 34], some patients in this study who reported that they drank, had 0 pg/mg in hEtG. There might be different reasons for this. Although hEtG has been proved to perform excellently in the cut-off level of 30 pg/mg, the performance in the lower ranges is less accurate [17]. hEtG can also be influenced by cosmetic hair treatments and thermal hair straightening tools, leading to false negative results [18]. This may not be known by the researcher and is only rarely reported [33], which was also the case in this study, and might be an explanation for the gender-related differences. However, another possible explanation could be underpowered statistical analyses in women. Also, hEtG cannot be analysed in men without, or with too short, hair, which is the explanation for the gender differences between the groups with analysable and non-analysable hEtG. Moreover, drinking pattern (such as seldom binge drinking vs. continuous moderate intake) can influence hEtG [17]. In this study, self-reported alcohol consumption was assessed with only one single question concerning weekly alcohol intake.

Since substrate mapping is not performed in first time RFA at our centre, it was not included in the study protocol. Measures of the magnitude of electrical impairment were thus not obtained in this study. Finally, only performed re-ablations within one year after the initial RFA were included in the analyses. There might have been patients on the waiting list for re-ablation occurring later than one year after the initial RFA. Hence, the actual number that had a re-ablation at some time could have been greater.

## Conclusions

In contrast to previous studies relying on self-reported alcohol consumption, this is, to the best of our knowledge, the first study investigating the association between an objective marker of alcohol consumption with cardiac biomarkers, echocardiographic measurements and re-ablation in patients undergoing RFA. hEtG ≥ 7 pg/mg in men was associated with higher cardiac biomarker concentrations, larger LA volumes and a higher rate of re-ablations, while no such differences were found in women. This implies that men with an alcohol consumption corresponding to an hEtG-value ≥7, have a higher risk for LA remodelling that could potentially lead to a deterioration of the AF situation.
